# Review: Fructose, the Sweet Culprit behind Nonalcoholic Fatty Liver Disease and Type 2 Diabetes

**DOI:** 10.2174/0115733998336827250114071627

**Published:** 2025-02-04

**Authors:** Abdullah Y. Otayf, Gaurav Agarwal, Muzaynah Al Khairat, Elham Ahmed Khormi, Hamzah Hassan Alnemi, Ahmad Ali Alhazmi, Khalid Abutawil, Shilpi Agarwal

**Affiliations:** 1 Department of Clinical Nutrition, College of Nursing and Health Sciences, Jazan University, Jazan, 82817, Saudi Arabia;; 2 Department of Pharmacy, PIET, Panipat, Haryana, India;; 3 Department of Nutrition Science, College of Nursing and Allied Health Sciences, Howard University, District of Colombia, Washington DC 20059, United States;; 4 Department of Nursing, Primary Health Care Center, Al-Khashabiya, Jazan, Saudi Arabia;; 5 Department of Clinical Nutrition, Prince Mohammed Bin Nasser Hospital, Ministry of Health, Jazan, Saudi Arabia;; 6 Department of Clinical Nutrition, King Faisal Medical City, Ministry of Health, Abha, Saudi Arabia;; 7 Department of Applied Sciences, Panipat Institute of Engineering and Technology, Panipat, Haryana, India

**Keywords:** Insulin resistance, nonalcoholic fatty liver disease, dietary fructose, type 2 diabetes mellitus, dietary sugars, non-alcoholic steatohepatitis, fructose

## Abstract

The composition of the diet has altered over the past few decades, with a significant increase in the consumption of dietary sugars, particularly fructose, which has risen more than tenfold. This elevated consumption of fructose and sugars is considered to be one of the major risk aspects for the emergence of obesity and other metabolic disorders. In this review, we discuss the correlation between insulin resistance and NAFLD (nonalcoholic fatty liver disease) due to dietary fructose intake. Type 2 Diabetes Mellitus (T2DM) is spreading quickly over the world and has epidemic proportions. The risk of both hepatic and metabolic abnormalities is increased by their connection, founded on shared metabolic risk factors such as obesity, (IR) Insulin Resistance, and an unhealthy standard of living. This review highlights the prevalence of coexisting T2DM (Type 2 diabetes) and NAFLD (Non-alcoholic Fatty Liver Disease) in the community. In summary, the key pathophysiological pathways and risk stratification algorithms for the onset of nonalcoholic fatty liver disease and type 2 diabetes have been covered.

## 1 INTRODUCTION

The correlation between T2DM (Type 2 Diabetes Mellitus) and NAFLD (Non-alcoholic Fatty Liver Disease) is becoming more common in clinical practice, particularly in Western countries [1]. Using diagnostic techniques like liver ultrasound, Magnetic Resonance Spectroscopy/Imaging (MRI), or Computed Tomography Scan (CT), the occurrence of NAFLD is anticipated to be 25% worldwide [2]. According to estimates, 10.5% of persons between the ages of 20 and 79 worldwide have diabetes mellitus [3], with over 90% having T2DM. Cardiovascular Disorders (CVD) rank among the top causes of death for these individuals, followed by complications connected to the liver (cirrhosis, carcinoma) [4, 5].

The main objective is to understand the metabolism of fructose in the liver which is distinct from glucose metabolism, and fructose excess consumption has been linked to insulin resistance, lipid accumulation, and inflammation [6-8]. Below is a biochemical breakdown of the key pathways and mechanisms:

### 1.1 Metabolism of Fructose in the Liver

Fructose is predominantly metabolized in hepatocytes (liver cells). Unlike glucose, fructose bypasses key regulatory steps, entering glycolysis without feedback control [9-12].

- Fructokinase (KHK) converts fructose to fructose-1-phosphate using ATP.
- Aldolase B cleaves fructose-1-phosphate into glyceraldehyde and DHAP (Dihydroxyacetone Phosphate).
- Glyceraldehyde is further phosphorylated into glyceraldehyde-3-phosphate, entering glycolysis (Fig. **1**).

### 1.2 Contribution to Insulin Resistance

- *De Novo* Lipogenesis (DNL): Excess fructose enhances acetyl-CoA production, promoting fatty acid synthesis *via* the Fatty Acid Synthase (FASN) pathway. These fatty acids are converted into triglycerides.
- Elevated triglycerides interfere with insulin signaling by inhibiting the Insulin Receptor Substrate (IRS) and Akt phosphorylation, leading to insulin resistance.
- Fructose increases hepatic glucose production by promoting glucose-6-phosphatase activity, exacerbating hyperglycemia [13, 14].

### 1.3 Lipid Accumulation (Hepatic Steatosis)

- Excessive fructose promotes DNL, accumulating triglycerides within hepatocytes, contributing to Non-alcoholic Fatty Liver Disease (NAFLD).
- Impaired β-oxidation occurs because fructose metabolism generates malonyl-CoA, inhibiting carnitine palmitoyltransferase-1 (CPT1), the enzyme liable for transporting fatty acids into mitochondria for oxidation [15, 16].

### 1.4 Inflammation in the Liver

- Excess lipids undergo lipotoxicity, triggering Endoplasmic Reticulum (ER) stress and oxidative stress [17, 18].
- The build-up of ROS (Reactive Oxygen Species) induces the activation of stress kinases like JNK and IKK, impairing insulin signaling [19-21].
- Accumulated lipids activate Toll-Like Receptors (TLRs), particularly TLR4, initiating pro-inflammatory cascades through NF-κB and promoting the inflammatory cytokines release like TNF-α, IL-6, and IL-1β [22, 23].
- Kupffer cells, the liver’s resident macrophages, are activated, further amplifying inflammation (Fig. **1**) [19, 24].

## 2 FRUCTOSE CORRELATION WITH NON-ALCOHOLIC FATTY LIVER DISEASE

A diet high in fructose can rapidly contribute to the development of nearly all fundamental disorders associated with metabolic syndrome [25]. This medical illness is characterized by excessive fat accumulation in the trunk region of the body, as well as elevated levels of sugar in the bloodstream or impaired ability to regulate glucose diabetes (Fig. **2**).

Fructose is believed to contribute to the development of NAFLD due to its metabolism in the liver. Most fructose consumed during meals is not digested in the intestine. Instead, it reaches the liver *via* the portal vein. The term NAFLD encompasses two conditions: NAFL (Non-alcoholic Fatty Liver), which is characterized by a fat content above 5% of the liver's tissue, and NASH (Non-alcoholic Steatohepatitis), which involves combined cell inflammatory infiltrate in addition to fat accumulation. NAFLD is majorly associated with metabolic syndrome and excessive food intake, and it can result in the development of liver cancer or liver cirrhosis, particularly Hepatocellular Carcinoma (HCC) [26]. The occurrence of NAFLD is approximately 26% in developed countries and has had a notable rise in the number of patients in recent times. Fructose is the primary ingredient that causes the liver to accumulate fat, leading to an increase in liver energy and the development of fatty liver cells. Retrospective studies suggest that there is a higher consumption of fructose in individuals with NAFLD, and this consumption may have an impact on the severity of liver fibrosis (Fig. **3**) [27].

Studies conducted on animals have demonstrated that diets with a high fructose content (70% of total calories taken) compared to diets with a higher glucose content result in noticeable fatty liver disease and evidence of liver cell destruction, as observed through histological examination and laboratory analysis [28]. The objective of this review is to gather the existing evidence that substantiates the significance of fructose consumption in the progression of liver fibrosis and life expectation in individuals with NASH and T2DM.

## 3 RELATIONSHIP BETWEEN FRUCTOSE AND INSULIN RESISTANCE

As previously stated, fructose causes distinct metabolic alterations in the liver compared to glucose and other carbohydrates by circumventing the primary regulatory processes in glycolysis, resulting in a fast buildup of subsequent intermediates. Fructolysis produces Free Fatty Acids (FFAs), which are then converted into storage Triacylglycerols (TGs) through esterification. Intracellular TG accumulation results in increased serine/threonine phosphorylation of insulin receptor substrate, leading to a decrease in insulin signaling [29]. Moreover, the presence of fructose intermediaries within the liver can modify the patterns of gene expression related to mitochondrial dysfunction, endoplasmic reticulum stress, and the initiation of liver inflammation. These characteristics are known to be connected to the progression of insulin resistance in the liver [30].

Research has demonstrated that diets containing high levels of fructose can cause IR in rats, canines, and humans. When rats were given a diet that replaced starch with fructose, they had an increase in fasting insulin resistance and hypertriglyceridemia. The severity of impaired hepatic insulin action was directly related to the amount of fructose consumed. Studies have examined the impact of consuming more fructose in humans. A meta-analysis of 29 trials found that consuming fructose, either in equal amounts to glucose or in higher amounts, leads to the development of IR in the liver in non-diabetic adults. This effect is observed in the short-term and does not affect insulin sensitivity in peripheral or muscle tissues.

Conventionally, Nonalcoholic Fatty Liver Disease (NAFLD) is defined as the existence of hepatic fat buildup affecting more than 5% of hepatocytes on histological examination or more than 5.6% on MRS or MRI. This definition is based on ruling out other causes of steatosis, such as chronic hepatitis B or C virus infection and everyday alcohol consumption (more than 30 g for males and more than 20 g for Females). Nonalcoholic Steatohepatitis (NASH) and Nonalcoholic Fatty Liver (NAFL) are two histologically and clinically distinct entities that are combined to form NAFLD. The bidirectional relationship between NAFLD and T2DM, which is symbolized by IR (Insulin Resistance), is the main topic of this review. The primary published data on the occurrence of chronic liver disease and its death rates in the diabetic community have been examined [31].

### 3.1 Which Term,“NAFLD” or “MAFLD”, is Correct?

The term “NAFLD” has its origins in 1980, when Jurgen Ludwig gave the illness its name based on a group of 20 patients who did not abuse alcohol but had the histological signs of alcoholic fatty liver disease. Even though a lot of time has gone by and our knowledge of the etiology and pathogenesis of this disease has advanced significantly, this “anti-definition” has not been updated since then [31].

By rebranding NAFLD as MAFLD (Metabolic dysfunction associated fatty disease), Eslam *et al.* offered a more useful and clinically grounded approach, permitting a distinct entity apart from the present label “non-alcohol” [32]. The existence of steatosis (assessed by imaging, blood biomarkers, and liver histology) linked to any one of the subsequent three criteria: body mass index status, *i.e.* overweight, type 2 diabetes, or metabolic syndrome, is the basis for the diagnosis according to the worldwide expert consensus statement [33]. The current data on the use of biomarkers and treatment options may be negatively impacted by the modification of the disease definition.

### 3.2 NAFLD Prevalence in the T2DM Population

According to Amiri *et al.*, 54% of NAFLD patients in their early stages also had T2DM [34]. After ruling out secondary causes, a different study found that, among patients with T2DM, 85.3% also had concurrently fatty liver in the US, of which 70% most likely had NAFLD [35]. In a sample of T2DM patients chosen at random, other researchers determined that 42.6% of them had hepatic steatosis in the US [36]. Africa had the lowest incidence (30%), whereas Europe and West Asia had the greatest percentages of 68% and 67%, respectively [37]. When combined, T2DM and NAFLD coexist and interact in most patients, which elevates the risk of more severe consequences (such as CVD (Cardiovascular disease, and hepatocellular carcinoma). According to meta-analysis data, which includes almost 300,000 people tracked over five years, people with NAFLD were twice as likely to experience an incident type of diabetes than people without the condition. Conversely, having T2DM raises the chance of developing cirrhosis, NASH (Non-alcoholic Steatohepatitis), or hepatocellular cancer [38].

## 4 NAFLD'S PATH PHYSIOLOGICAL MECHANISMS

### 4.1 Genetic Factors

A significant body of evidence from the scientific community provides credence to the idea that fatty liver disease is a hereditary manifestation. An increased likelihood of developing the condition has been seen in first-degree relatives of patients who have been diagnosed with NAFLD. Glucokinase Regulatory Protein (GCKR), Membrane Bound O-Acyltransferase Domain Containing 7 (MBOAT 7), Transmembrane 6 Superfamily member 2 (TM6SF2), and Patatin-like phospholipase Domain-dontaining protein 3 (PNPLA 3) are some of the genes that have been linked to the hepatic lipid metabolism. These genes are well-known and widely recognized [39].

Furthermore, it was found that the obesity-related gene (FTO) and fat mass were related to a higher risk of obesity (and likely NAFLD as well) [40]. Increased expression of the FTO variant effector genes, Iroquois homeobox (IRX) genes 3 (IRX3) and 5 (IRX5), causes brown adipocytes, which dissipate energy, to transform into white adipocytes, which store energy. A higher fat diet is linked to an overexpression of FTO in hepatocytes, which encourages the development of Nonalcoholic Fatty Liver Disease (NAFLD), according to data from animal models [41].

### 4.2 Insulin Resistance (IR)

When fasting, the liver is essential to the metabolism of glucose because it releases glucose into the bloodstream for use by other organs. Insulin controls the hepatic synthesis of glucose and affects the glycogen synthesis/lysis and Gluconeogenesis (GNG) processes [42]. Following a meal, blood glucose rises, and incretin hormones, primarily GLP-1 (glucagon-like protein) and GIP (gastric inhibitory polypeptide), increase secretion of insulin. As a restraint on the rise in plasma glucose, insulin is injected into the liver *via* the portal vein, where it inhibits GNG. Reduced insulin secretion causes insulin levels to become insufficient to combat hepatic ischemia (IR), which results in a loss of GNG and glycogenolysis inhibition and an excess of glucose synthesis. Although insulin's capacity to regulate GNG is compromised in obese individuals, elevated insulin secretion frequently results in postprandial hepatic glucose and normal basal production rates [43]. However, individuals with Type 2 Diabetes (T2DM) produce more hepatic glucose due to a reduction in insulin secretion [44]. The IR in adipose tissue and its modified inhibition of lipolysis have an indirect influence on the metabolism of glucose in the liver. As a result, more free fatty acids are released, which are then transported to the liver and serve as the GNG's substrates. Some genes, like the Insulin Receptor Substrate-1 (IRS-1), ectoenzyme nucleotide pyrophosphate phosphodiesterase 1 (ENPP1 121 Gln), and 972 Arg polymorphism, which reduces the action of protein kinase B (AKT) in hepatocytes of the insulin receptor, define an interesting association between NAFLD and IR. Liver damage is predisposed to this by pathophysiological mechanisms, which results in decreased hepatic insulin signaling.

## 5 DIAGNOSIS

Type 2 diabetics are likely to develop non-alcoholic steatohepatitis and progressive fibrosis, according to all of the recommendations. A 2016 guideline from the European Association for the Study of the Liver states that screening with liver enzymes and/or the US should be done on all patients with metabolic syndrome or obesity, especially those with type 2 diabetes [45]. The American Association for the Study of Liver Diseases guidelines, on the other hand, state that cases of NAFLD or NASH development should only be considered “with an increased index of suspicion” [46]. The suggestion is based on the fact that there is no solid evidence on the diagnosis, prognosis, or treatment plan. Noninvasive techniques such as vibration-controlled transient elastography (VCTE), FIB-4, and NFS were also suggested by the AASLD for the development of fibrosis risk stratification. According to recent evidence, patients with type 2 diabetes can benefit financially from NAFLD screening and risk-stratification routes [47]. Based on the available data, the American Gastroenterological Association suggests threat stratification for advanced fibrosis and lean people with type 2 diabetes, as well as NAFLD screening [48]. Up to 20% of people with NAFLD progress to NASH, which is the next step in the disease's natural progression. According to histology, it is described as having more than 5% hepatic steatosis along with ballooning, an indication of hepatocyte destruction, and lobular inflammation, either with or without fibrosis. The Steatosis, Activity, and Fibrosis (SAF) score is an additional metric utilized to evaluate the degree of fibrosis, activity, and stage. One of its primary benefits is that it provides a realistic description of the three histological characteristics mentioned above, allowing for the classification of certain unique subgroups (such as patients with fibrosis and steatosis but no cell damage) [49].

Increased fructose consumption such as, High-fructose Corn Syrup (HFCS) and sucrose, has become a significant public health concern due to its links to metabolic disorders. Below is a detailed examination of its implications, as well as potential policy, dietary guidelines, and interventions to mitigate its impact [50-55].

## 6 PUBLIC HEALTH IMPLICATIONS

Excessive fructose consumption has been implicated in the rise of various non-communicable diseases (NCDs) [56].

Metabolic disorders like obesity and fructose promote lipogenesis andincreasing fat storage, leading to weight gain. Insulin resistance induced by fructose in type 2 diabetes elevates the risk of hyperglycemia, diabetes onset, and NAFLD [57-60]. The accumulation of lipids in hepatocytes increases the prevalence of NAFLD, which can lead to NASH (Non-Alcoholic Steatohepatitis), fibrosis, and cirrhosis [61, 62].

Cardiovascular risk factors, such as dyslipidemia, are promoted by fructose, which elevates triglycerides, LDL cholesterol, and fatty liver, which are key contributors to cardiovascular diseases. Hypertension: Uric acid generated from fructose metabolism contributes to endothelial dysfunction and increases blood pressure [63-66].

### 6.1 Current Dietary Guidelines and Recommendations

Several public health agencies recommend limiting added sugar intake, including fructose, to prevent metabolic diseases [67, 68].

- WHO Recommends encouraging added sugars to <10% of total daily energy intake and advises reducing it to 5% for additional health benefits [69].
- American Heart Association (AHA) suggests a daily added sugar intake limit of 25 g (6 teaspoons) for women and 36 g (9 teaspoons) for men [70, 71].
- Dietary Guidelines for Americans (DGA) encourages limiting added sugars to <10% of calories per day for those aged 2 and older [72-74].

These guidelines emphasize consuming whole foods (*e.g.*, fruits, vegetables, whole grains) over sugar-sweetened beverages (SSBs) and processed foods [68, 75, 76].

### 6.2 Potential Interventions and Strategies to Reduce Fructose Consumption

- Taxation of Sugar-sweetened Beverages (SSBs): Many countries have implemented SSB taxes (*e.g.*, Mexico, UK) to reduce the consumption of sugary drinks, which are a significant source of fructose. Evidence shows that SSB taxes lead to a reduction in sales and consumption, particularly among vulnerable populations, including children [77].
- Product Reformulation: Governments and industry partnerships encourage the reformulation of products to reduce added sugars, including fructose and HFCS, in processed foods.
- Nutritional Labeling and Public Awareness Campaigns: Front-of-package labels (such as “high in sugar” warnings) inform consumers and encourage healthier choices. Public health campaigns raise awareness about the risks of excessive fructose and sugar consumption, promoting whole foods over processed options [78].
- Behavioral and Lifestyle Changes: These include dietary education, teaching individuals to read food labels, and avoiding products with high added sugars. Substitution Strategies: Encouraging the consumption of whole fruits (which contain fiber and reduce fructose absorption) over fruit juices and sodas [79].

## 7 TREATMENT

### 7.1 Non-pharmacological Management

The main therapeutic goal for NAFLD is weight loss, according to an increasing amount of data. A 10% decrease in body weight was linked to fibrosis regression, as demonstrated by this dose-response relationship. Regretfully, less than 5% of the patients, in the aforementioned trial lost weight in 70% of them, primarily due to inadequate adherence and compliance with weight loss suggestions [80].

#### 7.1.1 Physical Exercise

Physical exercise has been demonstrated to diminish hepatic steatosis in NAFLD patients despite limited or no weight loss. This is likely because, as evidenced by rodent studies, it reduces the levels of the lipogenic enzymes fatty acid synthase and acetyl-co-enzyme. Nonetheless, there is proof that individuals who engage in physical activity for more than 2.5 hours per week or who increase their level of activity by more than 60 minutes per week over three months observe improvements in their liver enzyme levels regardless of their weight loss. Furthermore, sustained exercise has been shown to have positive effects on the primary risk factors for fatty liver, like visceral adiposity and type 2 diabetes [81].

#### 7.1.2 Diet

Calorie-free diets have been explored as potential nonpharmacological treatments for Non-alcoholic Fatty Liver Disease (NAFLD) since, as previously indicated, obesity and overweight are key risk factors and contributors to the development of NAFLD. Regardless of the macronutrient composition, data indicate that a decrease of 500-1000 kcal/ day results in suppression of endogenous glucose synthesis and reduction in IHTG and hepatic IR in the first 48 hours [51]. It raises muscle insulin-mediated glucose absorption over the long term (11 weeks). There is more and more data to support the advantages of the Mediterranean diet. This diet consists of whole grains, nuts, and an abundance of fruits, vegetables, and legumes. It promotes eating more fish and shellfish instead of processed and red meat. The anti-inflammatory and antioxidant properties of the Mediterranean diet, which include polyphenols found in fresh fruits, vegetables, nuts, olive oil, and vitamins, also have several advantages [82]. The dietary inflammatory index is a helpful tool for determining if a food has pro or anti-inflammatory qualities [83]. The maximum proinflammatory dietary pattern is 7.98 on the DII score, while the maximum anti-inflammatory dietary pattern is −8.87. These factors may account for these diets' advantages. Results from a low randomized trial with biopsy-verified NAFLD patients showed improvements in insulin sensitivity (IS) and a reduction in hepatic steatosis (measured by MRS). Furthermore, it is known that people with established diabetes who adhere to a Mediterranean diet have better glycemic control and a lower risk of cardiovascular disease [84].

### 7.2 Pharmacological Treatment

For patients who link T2DM and NAFLD, pharmacological medications that enhance IS may be an option because of the impact of IR on hepatic and adipose tissue. Furthermore, it is anticipated that certain diabetes medications that cause weight loss will also ameliorate T2DM and NAFLD.

Metformin is typically the first pharmaceutical line medication prescribed to individuals with type 2 diabetes [85]. Its advantages on IR include promoting muscle glucose absorption and lowering the GNG rate, making it a viable treatment for NAFLD. Metformin recovers the biochemical panel by lowering liver enzymes, IR, and BMI, according to data from a systematic study having patients with NAFLD. Metformin combined with lifestyle modification helps individuals with NAFLD and T2DM lose weight with a mean of 4.3-7.9% of their starting body weight, which reduces hepatic IS and enhances glycemic control. Sadly, metformin has little effect on the histological component of NAFLD [86].

Thiazolidinediones raise IS and have the potential to be used as a NAFLD therapy. The PPARγ (peroxisome proliferator-activated receptor gamma), which is rare in muscle and abundant in adipocytes, is agonistically activated by pioglitazone. By encouraging TG storage, improving the transcription of important lipogenesis-related genes, and enhancing the inhibitory effect of insulin on lipolysis, the activation of these nuclear receptors stimulates adipogenesis [87].

Pioglitazone therapy has been shown to raise plasma adiponectin levels in individuals with T2DM and NAFLD [88], which lowers IHTG and improves glycemic control. Regarding NASH, results also indicate that thiazolidinedione medication was linked to the improvement of any stage of fibrosis. While thiazolidinediones are beneficial for NAFLD and NASH, their use is currently restricted due to adverse effects, particularly an increase in weight and an increased risk of bone fracture [89]. The gut secretes GLP-1, an incretin hormone that decreases hunger and food intake, delays stomach emptying, inhibits glucose-dependent glucagon secretion, and increases insulin synthesis and release [90]. Due to the action of the enzyme Dipeptidyl Peptidase 4 (DPP4), which cleaves the GLP-1 molecule, its half-life is brief.

Individuals with T2DM and NAFLD who received treatment with canagliflozin, dapagliflozin, or empagliflozin had lower HBA1c values as well as lower IHTG and certain liver enzyme levels [91-93]. Furthermore, in a small cohort of 63 patients having T2DM and NAFLD, dapagliflozin demonstrated improvements above placebo in improving fibrosis and liver steatosis assessed with TE [94]. The betterment of the hepatic lipid content is mostly a result of weight loss because neither SGLT 2 inhibitors nor GLP-1 RAs are expressed in the liver. It is anticipated that the benefits of NAFLD will increase with the coupling of GLP-1RAs with SGLT2i [95, 96].

### 7.3 Procedures for Treating Obesity

Surgical operations remain a last resort for patients with obesity, T1DM, T2DM, and NAFLD who did not observe improvement in their liver despite lifestyle modification and medication treatment. Patients with a BMI between 30 and 39.9 kg/m^2^ who do not meet their glycemic targets, lose weight over time, and improve their comorbidities may also find metabolic surgery to be an option [97, 98].

Hepatic steatosis and the metabolic syndrome appeared to improve a year following bariatric surgery, according to clinical data from patients who were obese and had hepatic steatosis. Steatosis decreased by 8.5% on average (CI: 4-10; *p* < 0.0001), most likely as a result of the substantial weight loss following bariatric surgery. It also evaluated the relationship between IR and the degree of preoperative steatosis and how much better it got following surgery. This finding may help identify obese patients who may benefit from metabolic surgery. Clinical data regarding the kind of surgical treatments showed a significant reduction in the NAS score at one year and five years following the procedure, particularly in patients who received gastric bypass (Roux-en-Y gastric bypass) as opposed to adjustable gastric banding [99]. Endoscopic Bariatric and Metabolic Therapies (EBMTs) are minimally invasive approaches to weight loss that have similar outcomes to bariatric surgery but with a lower risk of side effects and at a cheaper cost [100, 101]. The three most popular procedures are aspiration techniques (AT), endoscopic sleeve gastroplasty (ESG), and intragastric balloon insertion (IGB) [102, 103].

## CONCLUSION

This review highlights the significant role of excessive fructose consumption as a key contributor to the growing prevalence of Non-alcoholic Fatty Liver Disease (NAFLD) and Type 2 Diabetes Mellitus (T2DM). Unlike glucose, fructose is metabolized predominantly in the liver, bypassing key regulatory steps in glycolysis. This unregulated metabolism promotes lipogenesis, leading to hepatic fat accumulation, oxidative stress, and inflammation, which are hallmark features of NAFLD. Simultaneously, fructose disrupts insulin sensitivity by impairing insulin signaling pathways, increasing insulin resistance, and promoting hyperinsulinemia, thereby exacerbating the risk of T2DM.

NAFLD is still underdiagnosed and under-evaluated despite its high occurrence, frequent correlation with type 2 diabetes, and high death rates (mostly from CV causes). This is primarily because there are no targeted public health initiatives in place. Those with type 2 diabetes have a higher occurrence of NAFLD than the overall population. NAFLD increases the development of CVD and doubles the risk of incident type 2 diabetes, depending on liver fibrosis and fat scores. Conversely, having T2DM raises the possibility that fatty liver would proceed to cirrhosis, NASH, or hepatocellular cancer. The higher intake of fructose, often like sugar-sweetened beverages and processed foods, creates a metabolic environment conducive to liver dysfunction and systemic glucose intolerance. In parallel, future research should focus on identifying precise molecular pathways through which fructose drives metabolic dysfunction, as well as evaluating the long-term efficacy of dietary interventions in clinical settings.

The cornerstone of therapy choices for people with both NAFLD and T2DM is lifestyle modification and weight loss, which not only improves glycemic management but also leads to metabolic health by easing the steatosis and fibrosis stage. In conclusion, the reduction of fructose intake represents a critical strategy for mitigating the burden of NAFLD and T2DM and improving overall public health. Early interventions targeting fructose consumption are imperative to curb the rising incidence of these interconnected metabolic disorders.

## Figures and Tables

**Fig. (1):**
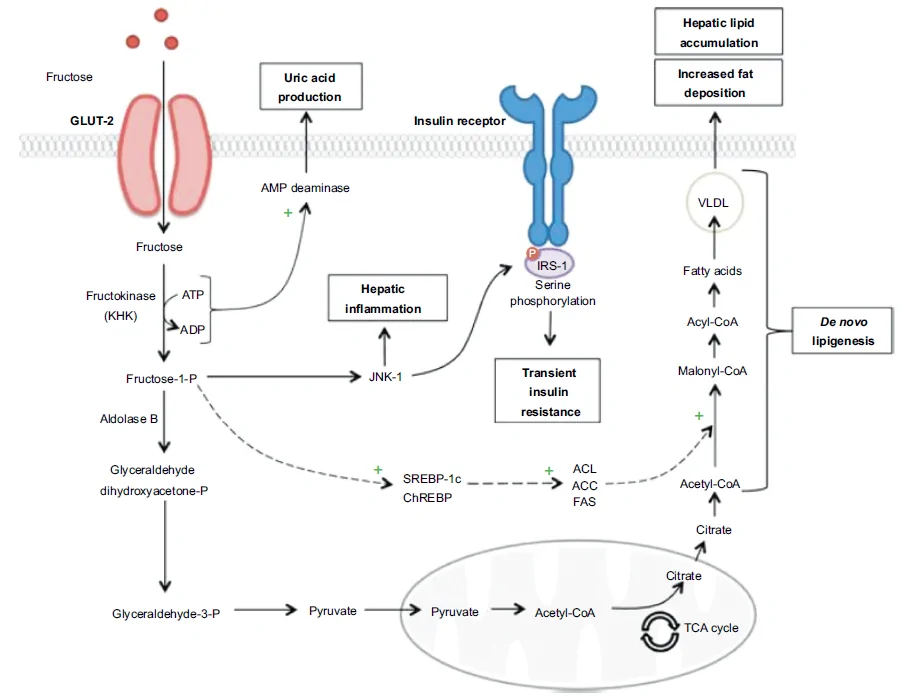
Mechanism of hepatic fructose metabolism.

**Fig. (2):**
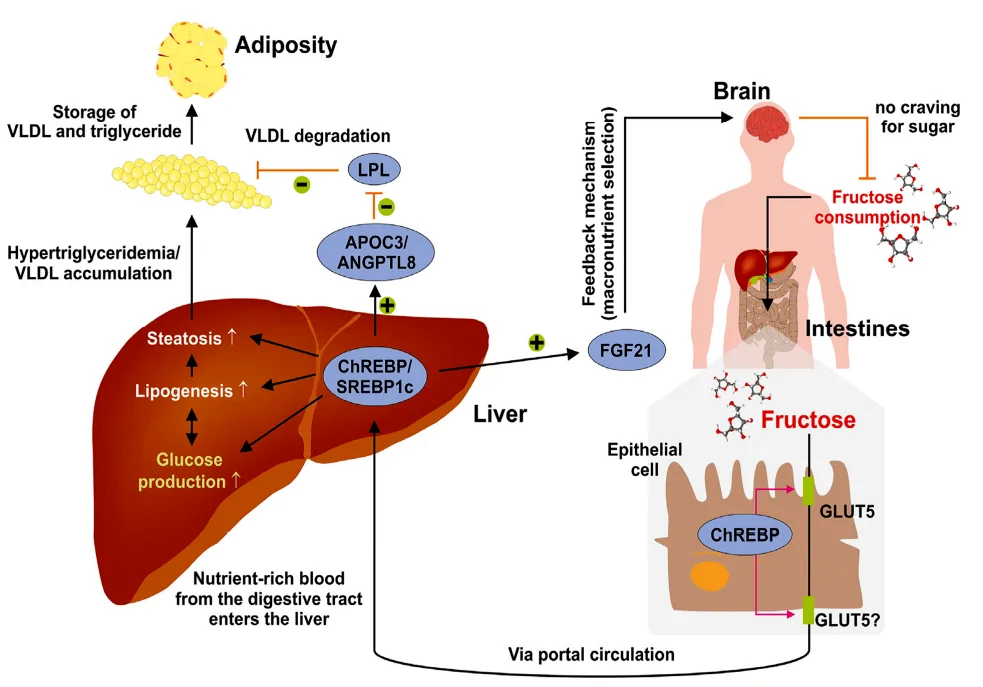
Role of fructose consumption in fatty liver disease progression: Mechanisms of lipid production and metabolic dysregulation "(adapted)" from Roeb and Weiskirchen, 2021, under the terms of the creative commons attribution license (CC BY).

**Fig. (3):**
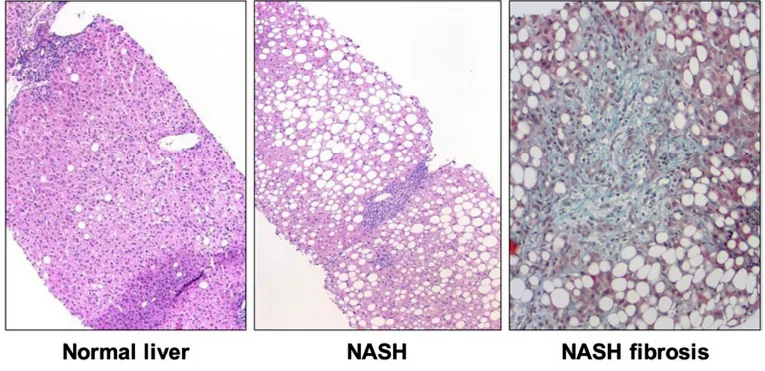
Histological analysis of tissue changes in non-alcoholic steatohepatitis (NASH): Comparison with normal liver and association with ballooning, steatosis, lobular inflammation, and fibrosis "(Adapted)" from Roeb and Weiskirchen, 2021, under the terms of the creative commons attribution license (CC BY).

## LIST OF ABBREVIATIONS

NAFLD: Non-alcoholic Fatty Liver Disease
T2DM: Type 2 Diabetes
NASH: Non-alcoholic Steatohepatitis
MRI: Magnetic Resonance Spectroscopy
CT: Computed Tomography Scan
CVD: Cardiovascular Disorders
APOC3: Apolipoprotein C-III
ANGPTL8: Angiopoietin
FGF21: Fibroblast Growth Factor 21
VLDL: Very Low-density Lipoprotein
NAFL: Non-alcoholic Fatty Liver
HCC: Hepatocellular Carcinoma
Ffas: Free Fatty Acids
Tgs: Triacylglycerols
IR: Insulin Resistance
GCKR: Glucokinase Regulatory Protein
PNPLA 3: Patatin-like Phospholipase Domain- Dontaining Protein 3
MBOAT 7: Membrane Bound O-Acyltransferase Domain Containing 7
TM6SF2: Transmembrane 6 Superfamily Member 2
FTO: Obesity-related Gene
IRX: Iroquois Homeobox
GNG: Gluconeogenesis
GLP-1: Glucagon-like Protein
GIP: Gastric Inhibitory Polypeptide
IRS-1: Insulin Receptor Substrate-1
ENPP1 121 Gln: Ectoenzyme Nucleotide Pyrophosphate Phosphodiesterase 1
AKT: Action of Protein Kinase B
SAF: The Steatosis, Activity, and Fibrosis
IS: Insulin Sensitivity
Pparγ: Peroxisome Proliferator-activated Receptor Gamma
